# Presence of phthalate derivatives in the essential oils of a medicinal plant *Achillea tenuifolia*

**DOI:** 10.1186/s40199-014-0078-1

**Published:** 2014-11-28

**Authors:** Azadeh Manayi, Mahdieh Kurepaz-mahmoodabadi, Ahmad R Gohari, Yousef Ajani, Soodabeh Saeidnia

**Affiliations:** Medicinal Plants Research Center, Faculty of Pharmacy, Tehran University of Medical Sciences, P.O. Box 14155–6451, Tehran, Iran; Institute of Medicinal Plants (IMP), Iranian Academic Centre for Education, Culture and Research (ACECR), Karaj, Iran

**Keywords:** *Achillea tenuifolia*, Compositae, Phthalate contamination, Acid treatment

## Abstract

**Background:**

Phthalate, esters of phthalic acid, are mainly applied as plasticizers and cause several human health and environment hazards. The essential oils of *Achillea* species have attracted a great concern, since several biological activities have been reported from varieties of these medicinal species. On the other side, due to the problems regarding the waste disposal in developing countries, phthalate derivatives can easily release from waste disposal to the water and soil resulting in probable absorption and accumulation by medicinal and dietary plants. As a matter of fact, although the toxicity of phthalate derivatives in human is well-known, food crops and medicinal plants have been exposing to phthalates that can be detected in their extracts and essential oils. *Achillea tenuifolia* (Compositea) is one of these herbaceous plants with traditional applications which widely growing in Iran.

**Finding:**

The plant root was subjected to hydro-distillation for 4 h using Clevenger type apparatus to obtain its essential oil before and after acid treatment. Both of the hydro-distilled essential oils were analysed by GC-MS method resulted in recognition of their constituent. Phthalate contamination as (1, 2-benzenedicarboxylic acid, bis (2-methylpropyl) ester (5.4%) and phthalic acid (4.5%), were identified in the first and second extracted oils, respectively.

**Conclusion:**

As a warning, due to the potential role of phthalates to cause reproductive toxicity, disturb of endocrine system and causing cancers, medicinal plants have to be considered through quality control for detection of these compounds.

## Findings

Regarding the recent published articles on probable pollution of medicinal plants and other natural medicines like marine algae to phthalate [1,2], finding a detection and even quantification method for phthalates, which can be accurate, fast and cost effective, is a considerable challenge particularly in standardization of herbal extracts and phytopharmaceuticals.

In fact, phthalates are the esters of phthalic acid and mainly used as plasticizers. They are manufactured by reacting phthalic anhydride with alcohols (ranged from methanol (C1) to tridecyl alcohol (C13)) in both straight and branching chains. Due to the toxicity concerns related to lower molecular weight phthalates (3–6 C), they are now being slowly replaced in the US, Canada, and European Union by high molecular weight phthalates (>6 C). The reason might be behind their higher permanency and durability in nature [2]. It is assumed that six million tonnes plasticizers are consumed every year, of which phthalates used in a large number of products including enteric coated pharmaceutical pills and supplements (as viscosity control agents), gelling agents, film formers, stabilizers, dispersants, lubricants, binders, emulsifying agents, and suspending agents [3]. These compounds interfere with endocrine systems in humans specially sex hormones and thyroids [4]. In addition, induction of inflammation, early puberty in girls, oxidative stress, asthma, and allergic symptoms were reported because of these compounds [5-7]. Literature review showed that these compounds could exhibit toxicity in liver, kidney, lung and testis in both animal and human [2,5]. Accumulation of phthalates may occur in a variety of herbal medicines especially those are growing up in water and rivers due to the exposure of plants’ roots to the polluted wastewater. Consequent exposure of animals and humans to phthalate by using polluted herbs, crops and vegetables is possible, since phthalates accumulate in plants [1].

*Achillea tenuifolia* is distributed in the north and north-west of Iran with small yellow flowers, woody based and several stems [8]. This plant has been used as traditional herbal remedies against sweating and bleeding along with regulation of menstrual cycle and reduction of heavy bleeding and pain [9]. The previous study revealed that the oil of the plant compromised of several monoterpenes and sesquiterpenes [9-12]. There is also a report on the phytochemical content of the root extract demonstrating the presence of tannins, sterols and terpenoids [13].

Recently, we reported high percentage of phthalate in a medicinal plant, *Lythrum salicaria* [14]. In continuing our research on detection of phthalate in medicinal and food plants, here we focused on detection of these compounds in the root oil of *A. tenuifolia.*

## Methods

### Plant material and isolation of essential oils

The roots of *A. tenuifolia* were collected from Qazvin province (1500 m above the sea level) in June 2011(No. 1624) deposited at the Herbarium of Institute of Medicinal Plants, Jahade-Daneshgahi (ACECR), Karaj, Iran.

Air-dried roots (200 g) were submitted to hydro-distillation in a Clevenger-type apparatus for 4 h, subsequently, 10 mL hydrochloric acid (Merck, Darmstadt, Germany) (1 N) was added to the residue of the root over night at room temperature and hydro-distilled again for 4 h. As a result of acid attendance in the mixture, hydrolysing procedure of glycosidic components was successfully facilitated. The oils after extraction were separately collected in screw capped glass vials and dried over anhydrous sodium sulphate (Merck, Darmstadt, Germany) and stored at 4°C until analyses.

#### GC-MS analysis

The essential oil was analysed by GC-MS method on a Thermoquest-Finnigan Trace GC-MS instrument (ThermoQuest, Manchester, UK) equipped with a DB-5 fused silica column (60 m × 0.25 mm i.d., film thickness 0.25 μm). The oven temperature was raised from 60°C to 250°C at a rate of 5°C/min and held for 10 min; transfer line temperature was 250°C. Helium was used as a carrier gas at a flow rate of 1.1 mL/min with a split ratio equal to 1/50. The quadrupole mass spectrometer was scanned over the 35–465 amu with an ionizing voltage of 70 eV and an ionization current of 150 μA. The compounds were identified by comparison of retention indices (RI, DB-5) with those reported in the literature and libraries [15-23].

## Results and discussion

**Figure 1:**
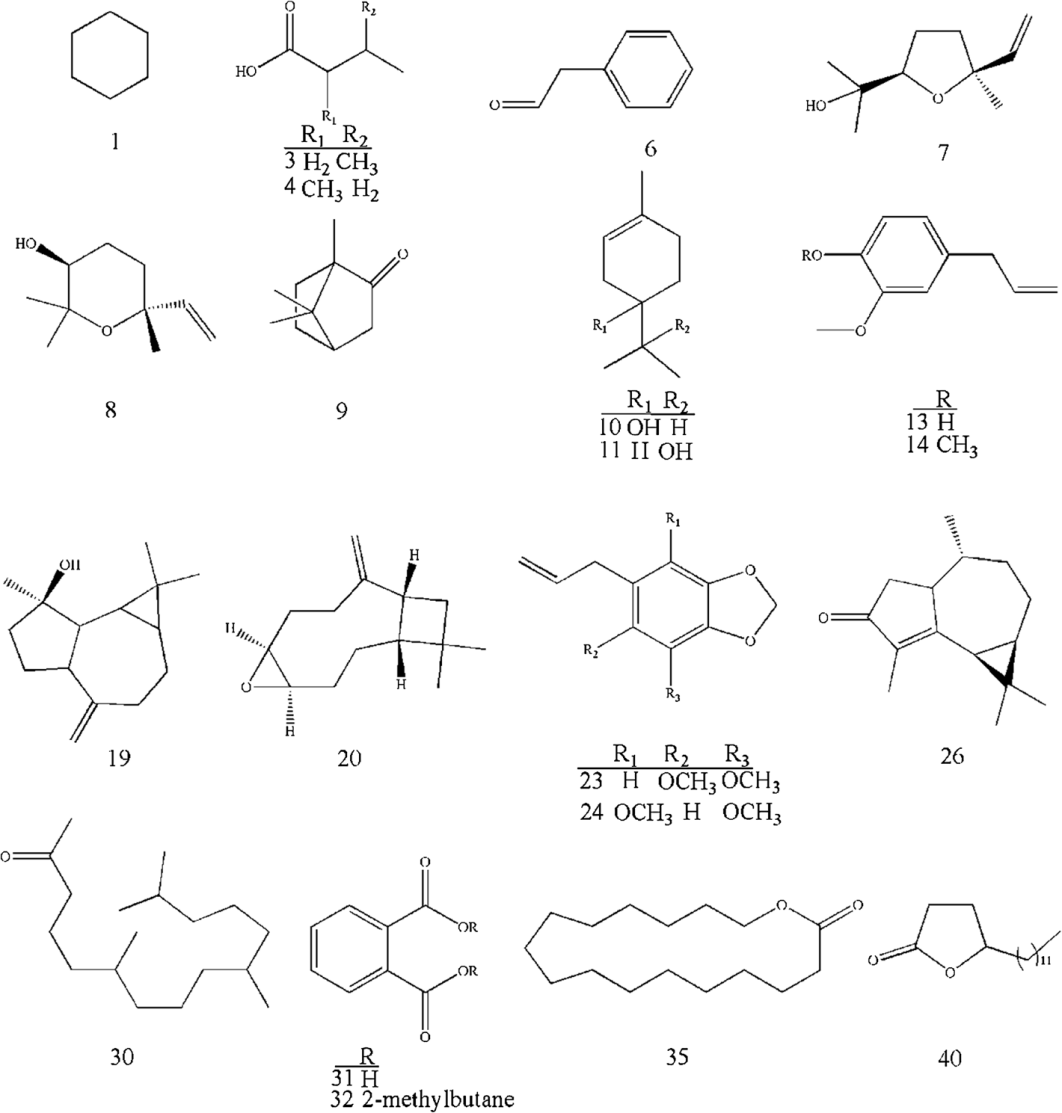
**Chemical structures of some identified components and phthalates (31 and 32) from the essential oils of** ***A. tenuifolia*** **root.**

The hydro-distillation of the root of *A. tenuifolia* resulted in extraction of the essential oils before and after acidic hydrolysis in extremely scarce amounts of colourless oils. In order to make sure about the sources of phthalate compounds in this study, no plastic container was used all through the procedure, and no solvent was used during extraction process except for hydrochloric acid that was purchased by analytical grade with no phthalate pollution. In addition, the solvents, used for injection of the samples to GC-MS, were injected alone to the chromatograph just before sample injection in order to detect probable contamination peaks. Taking together, any phthalate peaks detected in this study would highly unlikely be originated from storage, extraction and analysis procedure. GC-MS analysis of the volatile oils revealed the presence of 24 and 29 volatile components in the oils before and after acid treatment, representing 95.3% and 94.2% of the total oils, respectively (Table 1). Palmitic acid (36.9%), 5-dodecyldihydro-2(3H)-furanone (14.9%) and pentadecanoic acid (5.7%) were detected as the major constituents of the untreated essential oil, while the major volatile aglycones were identified as iso-valeric acid (24.9%), palmitic acid (15.8%), cyclohexane (13.3%), cyclohexadecanolide (7.2%) and 5-dodecyldihydro-2(3H)-furanone (6.1%) in the hydrolysed oil. Chemical structures of the identified compounds are illustrated in the Figure 1. However, in the previous study on the aerial parts of this plant, monoterpenes were characterized as the major constituents of the oil [5,6]. Regarding the present results, palmitic acid and 5-dodecyldihydro-2(3H)-furanone were dominant in both volatile oils. The most considerable point found among the identified compounds is the presence of phthalate contaminations (compounds 31 and 32 in Figure 1) in both oils identifying as 1,2-benzenedicarboxylic acid, bis (2-methylpropyl) ester (5.4%) in the oil before acid treatment and phthalic acid (4.5%) in the oil after acid treatment. Presence of phthalic acid in the oil after acid treatment probably attributed to the hydrolysis of its derivatives during acid treatment.

**Table 1:** **Percentage composition of the essential oils obtained from** ***A. tenuifolia*** **root before and after acidic hydrolysis**

**No.**	**Identified compounds**	**KI**	**RT**	**Percentage (%)**	
				**Content** ^**a**^	**Content** ^**b**^
1	Cyclohexane	752	5.12	-	13.3
2	n-octane	900	6.68	-	0.4
3	Iso-valeric acid	976	8.15	-	24.9
4	2-methyl butanoic acid	978	8.19	-	0.6
5	n-decane	1098	11.16	0.6	1.2
6	Benzene-acetaldehyde	1146	12.43	-	0.2
7	Linalool oxide (cis) furanoid	1176	13.3	-	0.4
8	Linalool oxide (trans) pyranoid	1191	13.73	-	0.3
9	Camphor	1245	15.23	0.3	-
10	Terpinene-4-ol	1273	16.15	0.5	-
11	Alpha-terpineal	1289	16.54	0.6	-
12	Dodecane	1297	16.8	-	0.5
13	Eugenol	1453	21.28	1	2.7
14	Methyl eugenol	1490	22.07	0.4	-
15	n-dodecanol	1493	22.15	0.4	-
16	n-tetradecane	1497	22.27	-	0.3
17	Pentadecane	1597	24.81	-	0.1
18	Dodecanoic acid	1649	26.04	2.6	-
19	Spatulenol	1687	26.99	2	-
20	Caryophyllene oxide	1690	27.07	2	-
21	Hexadecane	1697	27.23	-	0.4
22	Tetradecanal (myristaldehyde)	1763	27.54	3.9	1.8
23	Dill apiol	1771	28.91	-	0.1
24	Apiol	1788	29.3	0.5	-
25	Tetradecanoic acid (myristic acid)	1842	30.95	4	1.9
26	Cyclocolorenone	1855	30.8	0.5	-
27	Octadecanal	1895	31.3	-	0.3
28	Hexadecanal	1897	31.7	0.3	0.4
29	Pentadecanoic acid	1902	32.28	5.7	4.5
30	6,10,14-trimethyl, 2-pentadecanone	1928	32.38	0.9	-
31^c^	Phthalic acid	1944	32.7	-	4.5
32^c^	1,2-benzenedicarboxylic acid, bis (2-methylpropyl) ester	1955	32.93	5.4	-
33	Hexadecanoic acid (palmitic acid)	2027	34.32	36.6	15.8
34	9-octadecanoic acid (oleic acid)	2048	34.38	9.7	-
35	Cyclohexadecanolide	2053	34.9	-	7.2
36	Ethyl stearate	2079	35.41	0.9	-
37	Docosane	2087	35.57	0.5	0.2
38	Ethylhexadecanoate	2091	35.65	-	0.4
39	Heneicosane	2145	37.72	-	0.5
40	5-dodecyldihydro-2(3H)-furanone	2150	37.92	14.9	6
41	Ethyl linoleate	2177	38.95	1.3	2.5
42	Nonadecanal	2226	40.3	-	2
43	Tricosane	2322	42.63	-	0.8
Hemiterpenoids				-	24.9
Monoterpenes				2.8	3.4
Sesquiterpenes				4.5	-
Phenylpropanoids				0.5	0.1
C_X_H_y_				1.3	17.8
C_x_H_y_O_z_				86.4	48
Phthalate contamination				5.4	4.5
**Total**				95.5	94.2

*KI*: Kovats Index on DB-5 with reference to n-alkanes injected after the oil at the same chromatographic conditions, *RT*: Retention Time, a: values of the percentages before acidic hydrolysis, b: values of percentage after acidic hydrolysis, c: phthalate derivatives contaminations.

Detection of the mentioned compounds in the oils revealed that these contaminations are able to absorb from water and soil into the plant root. The plant, employed in this study, was gathered from a mountainous region near a seasonal river, which was surrounded by lots of disposed plastics and water bottles. Therefore, the source of contamination would most probably be polluted water particularly, regarding the point that we reduced the probable external contamination during storage, extraction and analysis procedure. Actually, these phthalate derivatives are widely used in plastic items, medical and pharmaceutical products, health care products, food containers, toys and paints. It seems that in Iran, the major sources of these compounds might be disposal plastics and chemical factories. Phthalate contaminations have previously been reported from the essential oils of the plants in several studies reported phthalate contaminations in the plants oils [14,24-27]. Exposure to phthalates during pregnancy produced serious adverse effects like miscarriage, low birth weight, and preterm birth trough induction of inflammation and oxidative stress [6]. Moreover, fetal exposure to phthalate is associated with behavioral and mental ability; for instance in the third trimester of pregnancy they caused neurogical problems in children even until 4–9 years [28]. Although finding such a toxic manmade group of compounds is not a new concern and they are now replaced in the USA, Canada, and European Union by other plasticizers, but there is a complicated situation in developing countries. In fact, U.S. Environmental Protection Agency (EPA) has current management plan that includes the following eight phthalates: dibutyl phthalate (DBP), diisobutyl phthalate (DIBP), butyl benzyl phthalate (BBP), di-n-pentyl phthalate (DnPP), di(2-ethylhexyl) phthalate (DEHP), di-n-octyl phthalate (DnOP), diisononyl phthalate (DINP), and diisodecyl phthalate (DIDP), of which, BBP, DEHP, and DBP cause the most toxicity to terrestrial organisms, fish, and aquatic invertebrates. Medical device assessments for DEHP have been developed by Food and Drug Administration (FDA), Health Canada Medical Devices Bureau and the European Union Scientific Committee on Medicinal Products and Medical Devices. They concluded that premature infants are the population most highly exposed to phthalates via these uses. Furthermore, The European Commission (2005) banned DEHP, DBP and BBP in all toys and childcare articles. Encouraging industry to move away from phthalates is future plan of EPA [29].

## Conclusion

Finding the phthalate esters in the essential oil of *A. tenuifolia* indicated that these toxic compounds, which have been used as the plasticizers in chemical and pharmaceutical industries, are able to be simply released into the water and soil and accumulate in the plants even in the medicinal species that are growing wildly in mountainous areas surrounded by lots of municipal solid wastes, disposed plastics and water bottles. Derivatives of phthalate esters are able to cause reproductive and developmental toxicity [1,26] regarding their chemical structures. The toxicity of phthalate esters have been well-documented demonstrating that different organisms and tissues of the human and animal bodies could be affected by them including kidney, liver, thyroid and testes [1,2,30]. Besides, they could sensitize eye, skin and mucus membranes in human [2]. Taken together, pollution of medicinal plants to phthalate esters in developing countries seems cause a major problem in human health area, which needs more attention in both quality control and standardization of herbal medicines as well as Food and Drug policies or strategies by Ministry of Health.
